# Queen-Driven Color Image Encryption Based on 2D-NECS Hyperchaos

**DOI:** 10.3390/e28070739

**Published:** 2026-07-01

**Authors:** Xuecheng Yang

**Affiliations:** School of Computer Science and Technology, Shenyang Institute of Engineering, Shenyang 110136, China; yungxc@126.com

**Keywords:** two-dimensional chaos, nonlinear exponential system, Queen-based permutation, indexed row–column diffusion

## Abstract

A color image encryption scheme is developed by integrating a two-dimensional nonlinear exponential chaotic system (2D-NECS), Queen-driven permutation, and indexed row-column diffusion. The proposed 2D-NECS generates highly sensitive pseudo-random sequences for constructing dynamic permutation indices and diffusion parameters. A Queen-driven traversal mechanism achieves multi-directional pixel scrambling and enhanced cross-channel coupling, while indexed row–column diffusion propagates local changes throughout the entire image. Experimental results show that the encrypted images exhibit uniform histogram distributions, low pixel correlations, and information entropy values close to the theoretical ideal. Moreover, differential, chosen-plaintext, and known-plaintext attack analyses verify the strong security of the proposed scheme. These results demonstrate that the proposed method provides effective resistance against various cryptographic attacks while ensuring accurate image reconstruction.

## 1. Introduction

Digital images are characterized by large data volumes, high redundancy, and strong inter-pixel correlations. With the rapid development of digital technologies and networked systems, images have become one of the primary carriers of information in various applications. Consequently, ensuring the security of digital images has become increasingly important for maintaining reliable information exchange. Since such data often contain sensitive or valuable content, data leakage may lead to privacy breaches, unauthorized access, or misuse of information. Therefore, protecting digital image data during storage and transmission is of paramount importance.

A widely adopted approach for safeguarding images is encryption [1]. Conventional cryptographic algorithms, such as the advanced encryption standard (AES), provide strong and well-established security guarantees for general-purpose data protection. In parallel, a variety of image-oriented encryption methods have been developed, including techniques based on neural networks [2,3,4], neurons [5,6], S-box [7], DNA operations [8], rotating dial model [9], deep learning [10], and chaos system (CS) [11,12,13,14,15,16]. Among them, chaos-based image encryption schemes (IESs) have attracted extensive attention due to their inherent properties.

The security mechanism of CS-based IESs differs fundamentally from that of conventional cryptographic algorithms. In CS-based IESs, the overall security of the encryption process relies heavily on the quality of the generated chaotic sequences and their interaction with the permutation–diffusion structure. Therefore, the randomness and dynamical complexity of chaotic sequences are not only theoretical properties, but also directly determine the resistance of the IES against practical attacks. Existing CS-based IESs have made significant progress. For instance, Verma et al. proposed a secure heterogeneous multi-source IES integrating image fusion and optical encryption for efficient storage and secure transmission of multi-source images [17]. Lin et al. developed an industrial IES based on a dual-memristor Tabu learning neural network, exploiting complex chaotic attractors to enhance image security [18]. Zhang et al. proposed an adaptive IES combining compressed sensing, multi-layer chaotic systems, and the semi-tensor product to achieve adaptive compression and efficient image encryption for IoT and cloud computing applications [19]. Wang et al. introduce a nonlinear Arnold-based coupled map lattice and employ protein-based partitioning and bit-level permutation [20]. Belazi et al. propose a CS-based pixel arrangement method followed by XOR diffusion for real-time protection in telemedicine scenarios [21]. However, existing CS-based IESs with insufficient complexity may fail to provide adequate security robustness under these constraints, highlighting the need for more reliable chaotic models and encryption mechanisms.

To overcome the shortcomings, this work develops an encryption framework that combines a two-dimensional nonlinear exponential chaotic system (2D-NECS) with a Queen-driven permutation mechanism, termed NECS-IES. The 2D-NECS is utilized to produce highly sensitive pseudo-random sequences, which are further transformed into permutation indices and diffusion parameters to ensure sufficient randomness during encryption. Based on these sequences, a Queen-inspired permutation strategy rearranges pixel positions through dynamically constructed label matrices, enabling cross-channel interaction and effectively disrupting spatial correlations. Meanwhile, an indexed row-column diffusion process is applied to spread local variations across the entire image, thereby enhancing diffusion performance. In addition, plaintext-dependent hash initialization is incorporated into key generation, which improves key sensitivity and avoids equivalent key issues. Through the joint design of chaotic sequence generation, Queen-based permutation, and structured diffusion operations, the NECS-IES achieves strong resistance against statistical and differential attacks while maintaining efficient computational performance, making it suitable for secure image transmission in resource-limited environments. The main contributions are as follows:

1. A two-dimensional chaotic sequence generator based on the 2D-NECS is designed, providing high complexity and strong sensitivity to initial parameters for secure key generation.
2. A Queen-based permutation mechanism guided by dynamically constructed label matrices is proposed, enabling flexible and efficient pixel scrambling across image channels.
3. An indexed row–column diffusion strategy is developed to achieve the rapid propagation of pixel changes, thereby improving resistance to statistical and differential attacks.

The remainder of this paper is organized as follows. Section 2 introduces the 2D-NECS and analyzes its properties. Section 3 presents the NECS-IES framework, including key generation, permutation, and diffusion processes. Section 4 evaluates the performance and security of the NECS-IES through various experiments. Finally, Section 5 concludes this paper.

## 2. Related Work

### 2.1. Construction of the 2D-NECS

The logistic map and sine map are two classical one-dimensional CSs, which can be expressed as [22]:

(1) $$x_{n+1}=\mu x_{n}(1-x_{n})$$

(2) $$x_{n+1}=\mu \sin(\pi x_{n})/4$$

where μ denotes the control parameter. $x_{n}$ and $x_{n+1}$ represent the system states at the n and n+1 iterations, respectively. These systems lose their chaotic behavior when μ∈[0,3.57) and μ∈[0,3.46). Although they are simple and easy to implement, their relatively narrow chaotic ranges limit their effectiveness in IESs. To overcome these limitations, various enhancement approaches based on coupling mechanisms have been proposed. For instance, Gao et al. introduced an exponential term into the sine–logistic CS to enrich its dynamics [23]. Wang et al. developed a three-dimensional CS by combining logistic, sine, and linear components [24], and further extended it using fractional-order calculus to enlarge the chaotic region [25]. Nevertheless, many of these designs still rely on linear combinations or basic algebraic operations, which restrict their nonlinear complexity.

To address these issues, a new 2D-NECS is constructed as follows:

(3) $$\left\{\begin{array}{c} x_{n+1}=((4-\mu )e^{x_{n}^{2}}+\mu e^{y_{n}(1-y_{n})})\mathrm{mod}\;1\\ y_{n+1}=((4-\mu )e^{y_{n}^{2}}+\mu e^{\sin(\pi^{2}x_{n})})\mathrm{mod}\;1 \end{array}\right.$$

where μ is the control parameter, and $x_{n}$ and $y_{n}$ denote the state variables at iteration n, while $x_{n+1}$ and $y_{n+1}$ correspond to the updated states. The symbol n indicates the iteration index. The mod can limit the parameter range within [0,1). To evaluate the chaotic performance of the 2D-NECS, its dynamical properties are examined through bifurcation analysis, phase trajectories, Lyapunov exponents, sample entropy, and NIST statistical tests. In addition, comparative studies are conducted with several existing CSs, including those proposed by Teng et al. [26], Liu et al. [27], and Maity et al. [28].

#### 2.1.1. Bifurcation Diagram

**Figure 1:**
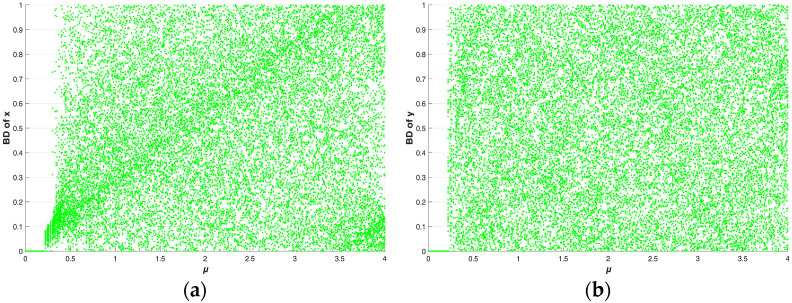
BD analysis: (**a**) $x_{n}$ and (**b**) $y_{n}$.

A bifurcation diagram (BD) illustrates how the dynamic states of a CS evolve as a control parameter changes, enabling the observation of transitions from regular periodic behavior to fully developed chaos [29]. As depicted in Figure 1, the 2D-NECS demonstrates rich and complex dynamical characteristics across a broad range of parameter values. This behavior indicates that the 2D-NECS can maintain chaotic properties over a wide interval. Consequently, the generated sequences and corresponding ciphertext exhibit approximately uniform statistical distributions, which enhances their ability to resist distinguishability-based attacks.

#### 2.1.2. Trajectory

**Figure 2:**
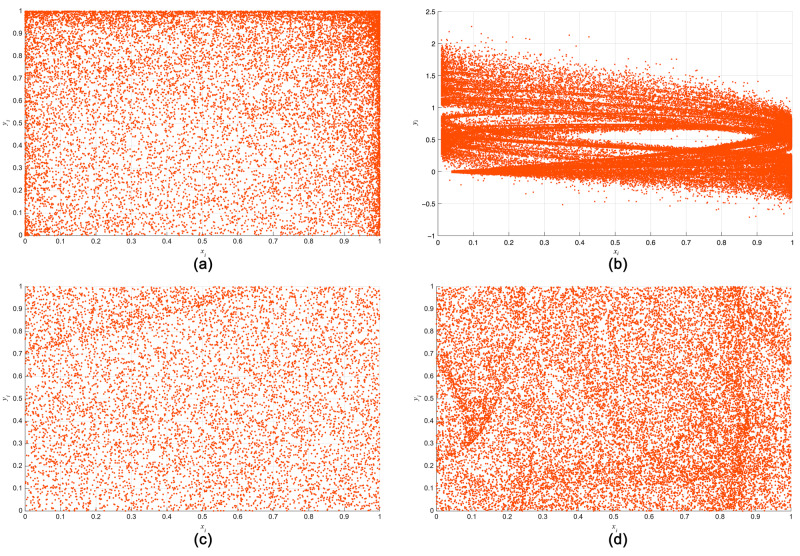
Trajectory distributions: (**a**) Teng et al. [26], (**b**) Liu et al. [27], (**c**) Maity et al. [28], and (**d**) 2D-NECS.

A trajectory diagram (TD) visually describes how a CS evolves in phase space and is a commonly used tool in the study of nonlinear dynamics and chaos theory [30]. As shown in Figure 2, the attractor generated by the 2D-NECS presents a well-distributed pattern across the phase plane, indicating a high level of uniformity compared with other CSs. This characteristic suggests that the 2D-NECS possesses enhanced ergodic behavior and improved randomness properties. Moreover, the absence of obvious clustering regions or structural gaps further demonstrates the superior phase-space coverage capability of the 2D-NECS.

#### 2.1.3. Lyapunov Exponent

The Lyapunov exponent (LE) measures the mean exponential separation rate of adjacent trajectories in phase space and is commonly adopted to describe the stability characteristics of dynamical systems. Generally, a positive LE implies strong sensitivity to initial conditions, whereas the existence of more than one positive LE indicates higher-dimensional complex dynamics, typically associated with hyperchaotic behavior. Moreover, larger LE values correspond to faster divergence of trajectories. The LE can be computed by [31]:

(4) $$\lambda =\underset{n\to \infty }{\lim}\frac{1}{n}\sum_{i=0}^{n-1}\ln\frac{d_{i}}{\delta_{0}}$$

where λ denotes the maximum LE, n represents the total number of iterations, and i is the iteration index. The term $d_{i}$ indicates the Euclidean distance between two nearby trajectories at the i-th step, while $\delta_{0}$ is the initial perturbation magnitude.

**Figure 3:**
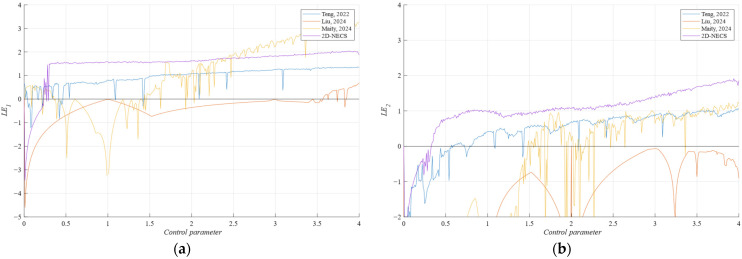
LE analysis: the (**a**) $LE_{1}$ and (**b**) $LE_{2}$ of Refs. [26,27,28] and 2D-NECS.

As tabulated in Figure 3, the 2D-NECS exhibits two positive LEs, confirming its hyperchaotic properties. Additionally, a comparative analysis between the 2D-NECS and several existing CSs [26,27,28] is conducted. The results reveal that the 2D-NECS possesses a wider hyperchaotic range and achieves larger LE values, indicating more intricate dynamical behavior. The larger positive LE values also imply a faster divergence of neighboring trajectories, which is beneficial for enhancing unpredictability. Therefore, the calculated LEs effectively reflect the strong sensitivity and divergence properties of the 2D–NECS.

#### 2.1.4. Cycle Length Estimation

**Figure 4:**
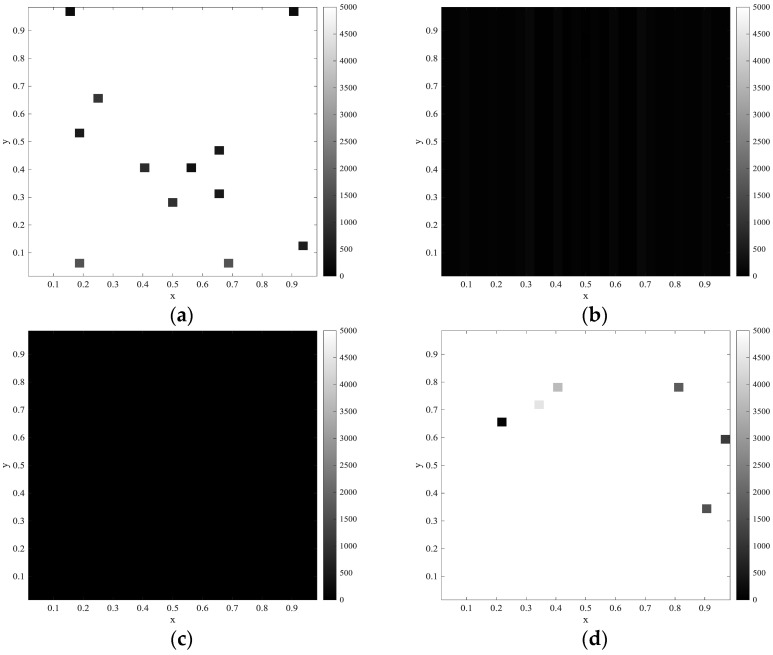
Estimations of the start time of the periodic mode in the chaotic sequence obtained for different CSs when the value of the control parameter is 3.9: (**a**) Teng et al. [26], (**b**) Liu et al. [27], (**c**) Maity et al. [28], and (**d**) 2D-NECS.

To evaluate the finite-precision degradation characteristics of the 2D-NECS, a cycle analysis was performed under 16-bit fixed-point arithmetic [32]. The initial conditions ($x_{0}$ and $y_{0}$) were uniformly selected from the interval [0.03125,0.96875], and each CS was iterated for 5000 steps. The starting time of the periodic mode was recorded for each initial condition pair, where larger values indicate stronger resistance to finite-precision degradation. As illustrated in Figure 4, the Ref. [27] and Ref. [28] systems exhibit extensive low-value regions, implying that periodic behavior emerges rapidly under finite precision. In contrast, both Ref. [26] and the 2D-NECS maintain high period-start values over most initial conditions. Moreover, the 2D-NECS exhibits a more uniformly distributed non-periodic region, demonstrating stronger resistance to dynamical degradation and longer effective cycle characteristics. These results indicate that the proposed exponential-coupling mechanism effectively enhances pseudo-randomness.

#### 2.1.5. State–Space Coverage

**Figure 5:**
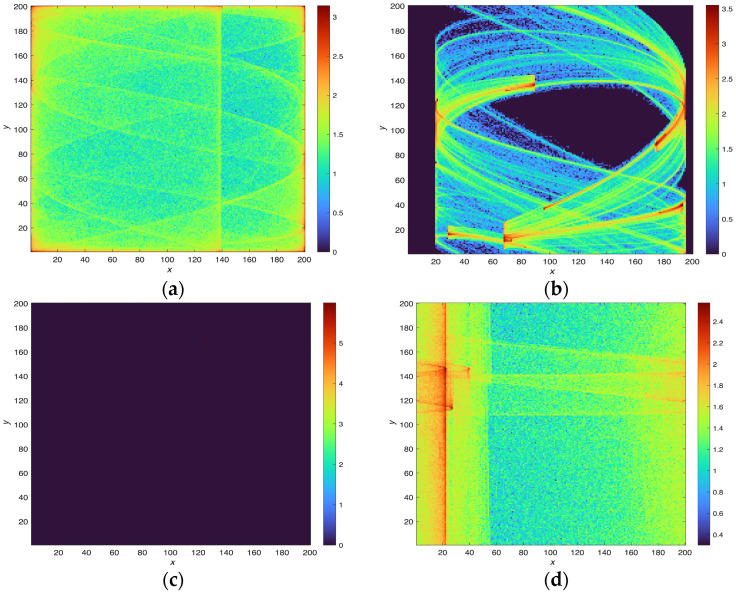
State–space coverage distributions of different CSs: (**a**) Teng et al. [26], (**b**) Liu et al. [27], (**c**) Maity et al. [28], and (**d**) 2D-NECS.

To further evaluate the pseudo-randomness and space-filling capability of the 2D-NECS, a state–space coverage analysis was performed and compared with several representative CSs. For each CS, $10^{6}$ state points were generated after discarding the transient iterations, and the phase space was uniformly partitioned into 100×100 grids. The visitation frequency of each grid was recorded to characterize the state–space occupation distribution. As illustrated in Figure 5, the 2D-NECS is capable of covering the entire phase space without leaving significant unvisited regions, indicating excellent ergodicity and space-filling capability. Compared with the CSs [26,27,28], the 2D-NECS exhibits a more uniform visitation distribution. These results demonstrate that the introduced exponential coupling mechanism effectively enhances the exploration ability of the chaotic trajectories, thereby improving the pseudo-randomness and cryptographic suitability of the generated sequences.

#### 2.1.6. Initial Value Sensitivity

**Figure 6:**
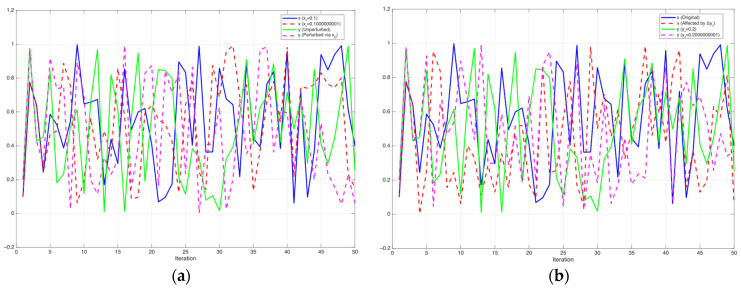
Sensitivity demonstration: (**a**) $x_{1}=0.1\to 0.1+10^{-10}$ and (**b**) $y_{1}=0.1\to 0.1+10^{-10}$.

Initial value sensitivity refers to the characteristic that arbitrarily small changes in initial conditions can produce significantly different evolution trajectories under the same system parameters, resulting in distinct long-term behaviors [33]. As the control parameter μ changes, Figure 6 presents the dynamic evolution of the 2D–NECS in terms of $x_{n}$ and $y_{n}$. For two almost identical initial settings, such as $x_{1}=0.1\to 0.1+10^{-10}$ or $y_{1}=0.1\to 0.1+10^{-10}$, the resulting trajectories quickly separate and develop into entirely uncorrelated sequences. This phenomenon clearly demonstrates that the 2D–NECS possesses strong dependence on initial conditions.

#### 2.1.7. Sample Entropy

**Figure 7:**
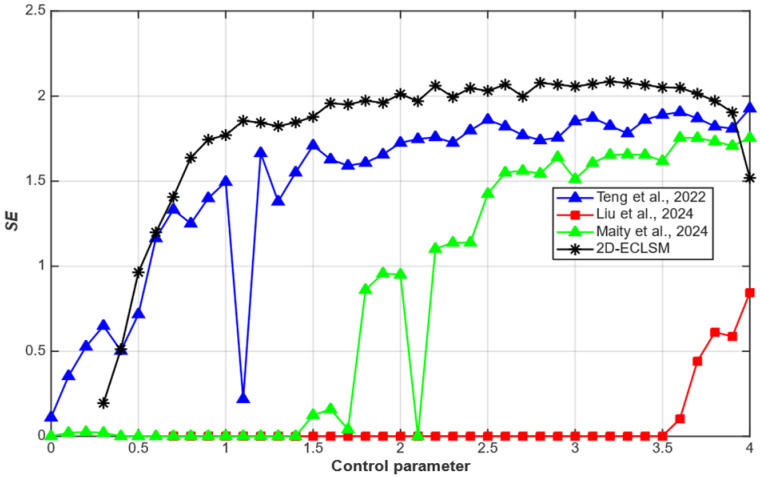
Comparison of SE with some well-established CSs [26,27,28].

According to [34], sample entropy (SE) is a quantitative indicator used to evaluate the irregularity and unpredictability of a time sequence. A higher SE value implies stronger randomness and a closer resemblance to ideal stochastic behavior, which is highly desirable for generating secure key streams. It should be emphasized that as the SE value of a CS increases, the predictability of the sequence correspondingly decreases. In this study, SE is defined as:

(5) $$SE(m,r,N)=-\log\frac{S}{P}$$

where m denotes the embedding dimension, r represents the tolerance threshold, and N is the length of the analyzed sequence. As illustrated in Figure 7, the 2D-NECS maintains consistently elevated SE values with only slight fluctuations. This behavior is attributed to the low matching probability between template vectors S and P under the Chebyshev distance criterion. The average SE value is higher than that of comparable CSs, demonstrating that the 2D-NECS is capable of producing sequences with enhanced randomness and greater dynamical complexity.

#### 2.1.8. NIST Test

The NIST SP800-22 suite consists of 15 statistical evaluations designed to identify deviations from randomness in binary sequences [11]. As tabulated in Table 1, all obtained p-values are above the threshold of 0.01, implying that none of the tests reject the null hypothesis of randomness. This outcome demonstrates that the sequences produced by the 2D-NECS possess strong statistical unpredictability and satisfy the required criteria for high-quality pseudo-random behavior.

### 2.2. Queen-Based Traversal Mechanism

#### 2.2.1. Traversal Mechanism

The Queen piece in chess provides a natural mechanism for describing multi-directional traversal on a two-dimensional lattice. It can move along horizontal, vertical, diagonal, and anti-diagonal directions, enabling flexible coverage of an n×n grid. Let each lattice point be denoted by a coordinate pair (x,y)∈{1,2,…,n}. The movement of the Queen can be modeled as a mapping $\left(x,y)\to (x^{\prime },y^{\prime }\right)$, controlled by a direction variable $d_{k}$ and a displacement distance $dist_{k}\in [-(n-1),n-1]$. The sign of $dist_{k}$ determines the movement orientation, while its magnitude specifies the step length. To ensure that the traversal remains within the lattice domain, a cyclic boundary condition is introduced:

(6) $$\left\{\begin{array}{c} x^{\prime }=((x^{\prime }-1)\mathrm{mod}n)+1\\ y^{\prime }=((y^{\prime }-1)\mathrm{mod}n)+1 \end{array}\right.$$

**Figure 8:**
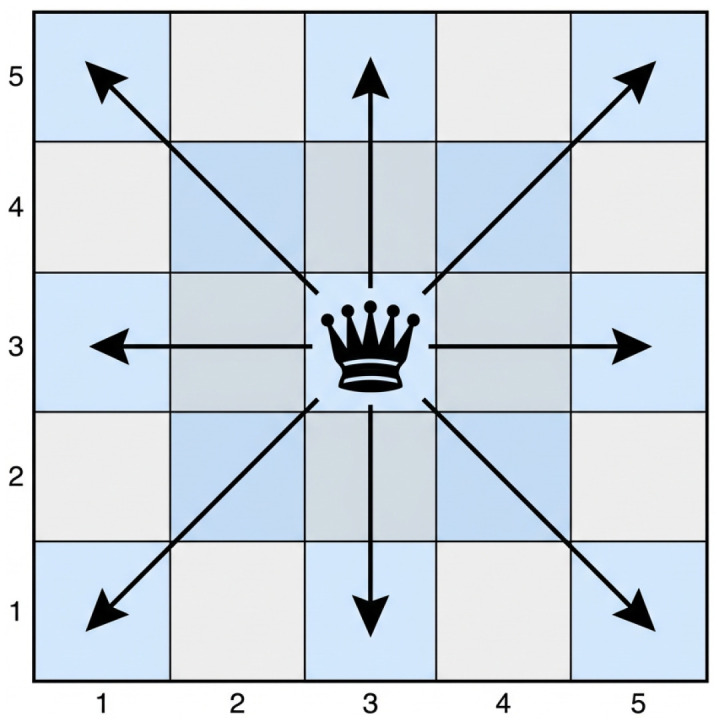
Illustration of the Queen-based traversal mechanism on a two-dimensional lattice.

Despite its flexibility, the traversal process exhibits several inherent limitations. The movement is sequential in nature and can be described as:

(7) $$\left(x_{k+1},y_{k+1})=f(x_{k},y_{k},d_{k},dist_{k}\right)$$

where each update depends on the previous state, potentially leading to redundant paths and uneven coverage. In addition, the deterministic movement rules may introduce structural regularity. Furthermore, explicit traversal requires additional mechanisms to avoid repeated visits, which increases computational complexity. To provide an intuitive understanding of the above traversal characteristics, a schematic illustration of the Queen-based movement on a two-dimensional lattice is presented in Figure 8, highlighting its multi-directional exploration capability.

#### 2.2.2. Three Modes of Traversal Mechanism

To overcome the above limitations, the traversal mechanism can be reformulated as a mapping problem defined over lattice indices. Let Q={Q(1),Q(2),…,Q(N)} be a permutation sequence over the index set {1,2,…,N}. For a lattice of size M×N, a mapping matrix $L\in R^{M\times N}$ is constructed to encode the interaction between row and column indices. Based on this formulation, three representative mapping strategies are defined as follows.

1. Symmetric coupling mode. This mode constructs the mapping through symmetric interaction between row and column indices. It is defined as:

(8) L(i,j)=Q(i)+Q(j)+δ×i×j

where δ is a control parameter. In this formulation, both indices contribute equally to the mapping value, forming a balanced coupling structure. The resulting mapping preserves global consistency across the lattice.

2. Cross-index mode. This mode introduces cross-index interaction by transforming the column index before applying the permutation. The transformed index is defined as:

(9) $$j^{\prime }=((j+Q(i)+\delta )\mathrm{mod}N)+1$$

and the mode is given by:

(10) $$L(i,j)=Q(i)+Q(j^{\prime })+\delta \times i\times j$$

By replacing Q(i) with $Q(j^{\prime })$, the direct correspondence between indices is disrupted, leading to increased nonlinearity and interdependence.

3. Feedback-based mode. This mode incorporates a feedback mechanism in which the column index dynamically affects the selection of the permutation index. The intermediate variable is defined as:

(11) t=(Q(i)+j+δ)modN)+1

and the mode is constructed as:

(12) L(i,j)=Q(i)+Q(t)+δ×i×j

In this formulation, the mode depends on both row and column indices through a feedback structure, resulting in a higher level of complexity coupling.

Moreover, to ensure a balanced distribution of mode values, the matrix L is further normalized. Let rank(⋅) denotes the ranking operation over all elements in L. The normalized mapping is defined as:

(13) L(i,j)=(rank(L(i,j))modN)+1

This operation redistributes the mapping values into a uniform range and guarantees that each index appears with equal frequency across the lattice.

#### 2.2.3. Cryptographic Advantages of Queen-Driven Traversal

Unlike conventional row–column permutation strategies, the Queen-driven traversal simultaneously exploits row indices, column indices, and dynamically generated permutation indices during the mapping construction process. Consequently, the generated traversal path is not restricted to a single horizontal or vertical direction but exhibits multi-directional coupling characteristics. Compared with standard Arnold algorithm, the proposed strategy introduces nonlinear index interactions and feedback-dependent path evolution, thereby producing more complex permutation trajectories.

Furthermore, the proposed traversal framework can be consistently applied to different color channels through the same mapping matrix, enabling the permutation process to preserve inter-channel coupling relationships. Unlike independent channel-wise permutation strategies, the Queen-driven traversal introduces stronger cross-channel dependency, thereby increasing the difficulty of exploiting statistical correlations among RGB components. This characteristic effectively enhances resistance against statistical recovery attacks and improves the overall confusion capability of the permutation stage.

## 3. NECS-IES Procedures

**Figure 9:**
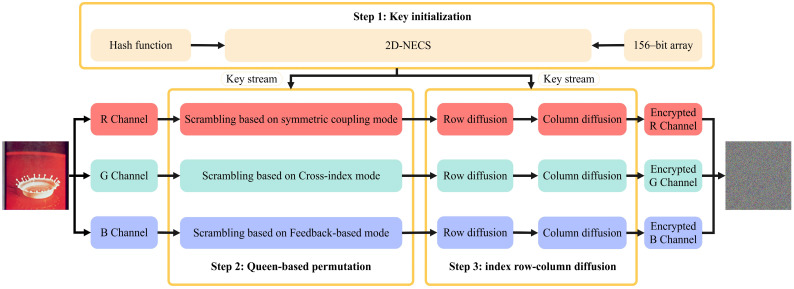
Structural illustration of the NECS–IES framework.

The NECS-IES employs the 2D-NECS due to its strong dependence on initial states and control parameters, which enables the generation of highly unpredictable pseudo-random sequences and enhances robustness against potential attacks. The overall structure of the NECS-IES is depicted in Figure 9. The framework is composed of three fundamental components: key construction, Queen-inspired permutation, and indexed row–column diffusion. During the key construction phase, the plaintext image and the 2D-NECS are jointly utilized to derive plaintext-dependent parameters and chaotic sequences required for encryption. Subsequently, these sequences are applied to guide the Queen-inspired permutation and indexed row–column diffusion operations. Together, these procedures make an effective and secure encryption process.

### 3.1. Key Initialization

The secret key set ks of the NECS-IES consists of two main components: a plaintext-related hash value $k_{\mathrm{HS}}$ and a randomly generated binary sequence $k_{R}$. The hash key $k_{\mathrm{HS}}$ is produced by applying the SHA–512 function to the input image, which introduces strong plaintext dependence and enhances the sensitivity of the NECS–IES to different image contents. As a result, the NECS-IES becomes highly responsive to variations in the input image. In addition, a 156-bit random binary sequence $k_{R}=\left\{bit_{1},bit_{2},\ldots ,bit_{156}\right\}$ is incorporated to regulate the parameters of the 2D–NECS, further strengthening the overall security.

Step 1: The plaintext image is first processed using the SHA–512 algorithm to generate a 128-character hexadecimal string $k_{\mathrm{HS}}$, where each character encodes 4 bits. Subsequently, 18 decimal values are derived according to:

(14) $$h_{\mathrm{HS}}=hex2dec(k_{\mathrm{HS}})$$

where the function hex2dec(⋅) converts the hexadecimal sequence $k_{\mathrm{HS}}$ into decimal form, followed by a modulo operation to constrain the results within the range [0,255].

Step 2: A 156-bit random binary sequence $k_{R}$ is generated based on the IEEE–754 double-precision floating-point representation. This sequence is then combined with the initial states (x and y) and the control parameter μ through Algorithm 1 [35]:

(15) $$x=\frac{\sum_{i=1}^{52}b_{i}2^{52-i}}{2^{52}}$$

where $b_{i}\in (0,1)$ denotes the i-th bit of the binary sequence $k_{R}$, and i is the corresponding index. The 52-bit mantissa structure ensures that the binary sequence can be accurately mapped into a real number within the interval (0,1).

Step 3: The updated parameters $\mu^{\prime }$, $x_{0}^{\prime }$, and $y_{0}^{\prime }$ obtained from Algorithm 1 are used as the initial inputs for the 2D–NECS. Prior to being utilized in permutation and diffusion operations, the chaotic sequences X and Y are further processed through normalization and index-based rearrangement, resulting in sequences $X_{\mathrm{int}}$ and $Y_{\mathrm{int}}$. These operations enhance the uniformity of the distributions and reduce potential bias, making the sequences more suitable for IESs. Finally, the 2D-NECS is iterated 3×M×N times to remove transient effects, and the resulting sequences are partitioned according to the requirements of subsequent encryption stages. The binary sequence $k_{R}$ is first transformed into an integer sequence and then normalized to produce parameters under double-precision representation.
**Algorithm 1.** Modification of 2D-NECS parametersInput: $k_{R}$, μ, $x_{0}$, $y_{0}$
Output: $\mu^{\prime }$
$,\;x_{0}^{\prime }$
$,\;y_{0}^{\prime }$
$1:\;\mu^{\prime }=\mu +((\sum_{i=1}^{52}k_{R}[i]\times 2^{i-1}/2^{52})\mathrm{mod}(4-\mu ))$$2:\;x_{0}^{\prime }=x_{0}+((\sum_{i=53}^{104}k_{R}[i]\times 2^{i-53}/2^{52})\mathrm{mod}(1-x_{0}))$$3:\;y_{0}^{\prime }=y_{0}+((\sum_{i=105}^{156}k_{R}[i]\times 2^{i-105}/2^{52})\mathrm{mod}(1-y_{0}))$

### 3.2. Encryption Process

The NECS-IES performed by Queen-based permutation and indexed row–column diffusion.

#### 3.2.1. Queen-Based Permutation

This process aims to rearrange the pixel positions through a Queen-based mapping mechanism. By integrating chaotic sequence $X_{\mathrm{int}}\in R^{M\times N\times 3}$ with a label-driven index transformation, the spatial distribution of pixels is significantly altered while preserving all pixel values.

Step 1: Image decomposition and permutation sequence construction. Given a color image $PI\in R^{M\times N\times 3}$, the chaotic sequence $X_{\mathrm{int}}$ is divided into three segments:

(16) $$\left\{\begin{array}{c} X_{1}=X_{\mathrm{int}}(1:N)\;\;\;\;\;\\ X_{2}=X_{\mathrm{int}}(N+1:2N)\;\;\\ X_{3}=X_{\mathrm{int}}(2N+1:3N) \end{array}\right.$$

where $X_{1}$, $X_{2}$, and $X_{3}$ are used to construct permutation sequences $Q_{k}\in \{1,2,3\}$ by sorting them, and each $Q_{k}$ defines a bijection $Q_{k}:\{1,2,3\}\to \{1,2,3\}$. $Q_{k}$ provides the ordering basis for Queen-based mapping construction.

**Figure 10:**
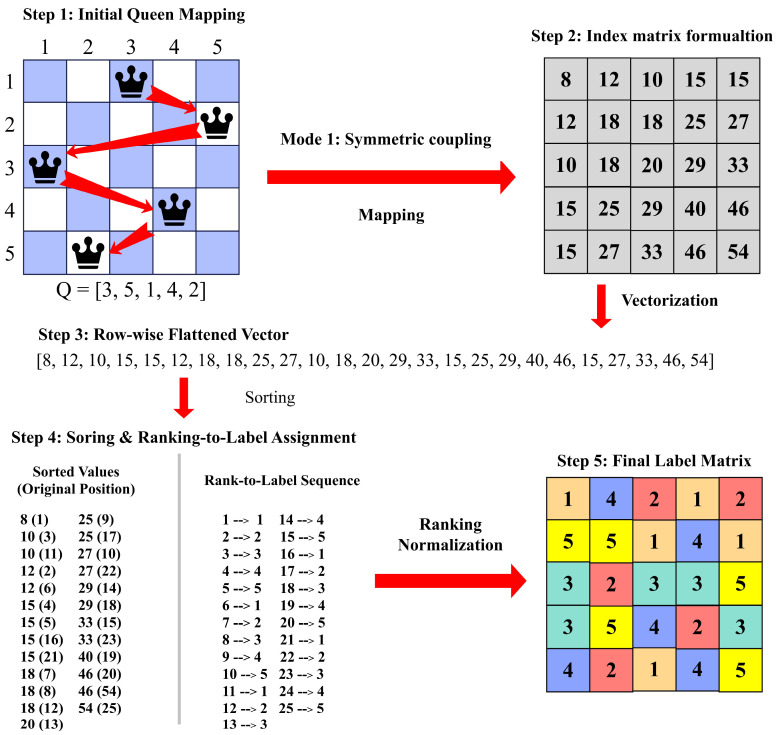
Symmetric coupling mode.

**Figure 11:**
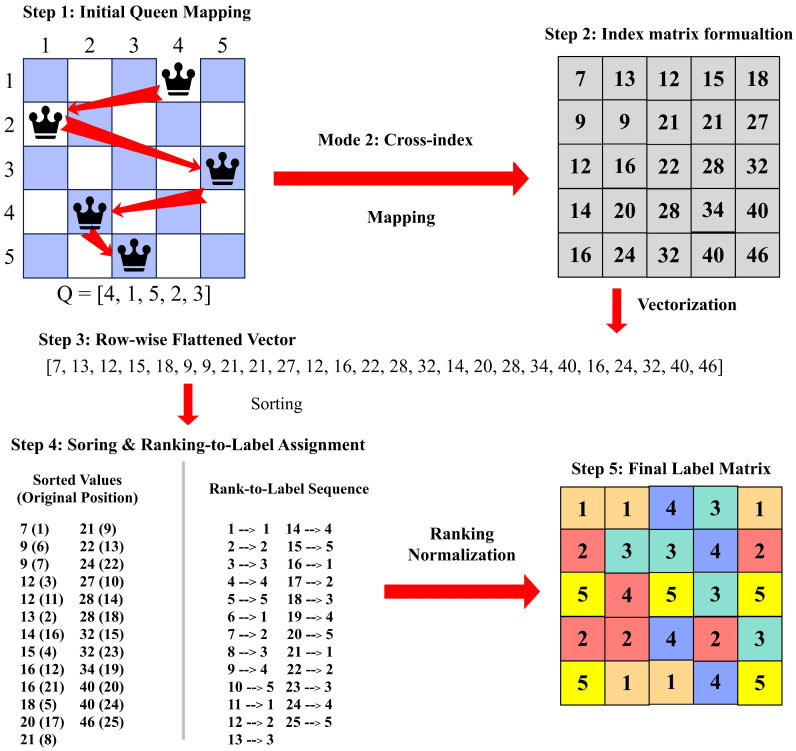
Cross-index interaction mode.

**Figure 12:**
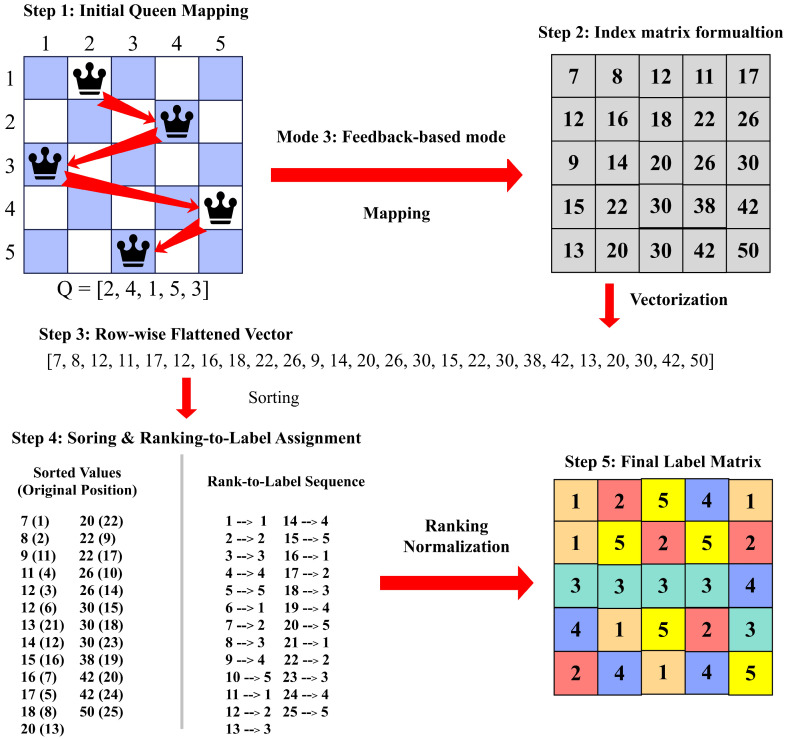
Feedback-based mode.

Step 2: Construction of Queen-based label matrices. For each permutation sequence $Q_{k}$, the label matrix $L_{k}\in R^{M\times N}$ is constructed. The mapping is derived from index interactions that emulate directional movements on the lattice. For each position (i,j), the intermediate value is defined as:

(17) index(i,j)=Q(i)+Q(φ(i))+δ×i×j

where φ(i) denotes a direction-dependent index transformation corresponding to different Queen-based movement patterns. To provide a clear illustration of the label construction process under different Queen-based movement strategies, three representative mapping modes are presented in Figure 10, Figure 11 and Figure 12, including symmetric coupling, cross-index interaction, and feedback-based transformation.

Step 3: Index transformation. To ensure a uniform distribution of mapping values, the intermediate matrix is converted into a label matrix $L_{k}(k=1,2,3)$ through Equations (8), (10) and (13). As a result, each row of $L_{k}$ forms a valid permutation, ensuring a uniform and balanced index distribution.

Step 4: Pixel rearrangement based on $L_{k}$. The permutation is performed by reassigning pixel positions according to the $L_{k}$. For each component, the rearrangement is defined as:

(18) $$PI_{R,G,B}^{\prime }(i,j)=PI_{R,G,B}^{\prime }(i,L_{k}(i,j))$$

This operation indicates that the pixel originally located at column $L_{k}(i,j)$ is relocated to position (i,j). Consequently, the permutation is carried out independently along each row, where the label matrix specifies the target column index for every pixel. These three modes lead to distinct label distributions, which enhance the diversity and randomness of the permutation process.

The process reorganizes the spatial distribution of pixels without altering their values, resulting in a pure position-based transformation. From a structural perspective, this relocation can be interpreted as a directional redistribution induced by the Queen-based mapping mechanism. The resulting matrices $PI_{R}^{\prime }$, $PI_{G}^{\prime }$, and $PI_{B}^{\prime }$ are taken as the three components of the permuted image.

#### 3.2.2. Indexed Row–Column Diffusion

This section implements the indexed row–column diffusion operation on the matrices $PI_{R}^{\prime }$, $PI_{G}^{\prime }$, and $PI_{B}^{\prime }$ based on the 2D-NECS.

Step 1: The following five subsequences are independently extracted from $Y_{\mathrm{int}}$ based on $k_{\mathrm{HS}}$ as follows:

(19) $$\left\{\begin{array}{c} CM_{D1}=Y_{\mathrm{INT}}(1:MN)\;\;\;\;\;\;\;\;\;\;\;\;\;\;\;\;\;\\ CM_{D2}=Y_{\mathrm{INT}}(MN+1:2MN)\;\;\;\;\;\;\;\;\;\;\;\;\\ CM_{D3}=Y_{\mathrm{INT}}(2MN+h_{\mathrm{HS}}(7)+1+2MN+h_{\mathrm{HS}}(7)+4M)\;\\ \begin{array}{l} CM_{D4}=Y_{\mathrm{INT}}(2MN+h_{\mathrm{HS}}(7)+h_{\mathrm{HS}}(8)+4M+1:\;\;\;\\ \;\;\;\;\;2MN+h_{\mathrm{HS}}(7)+h_{\mathrm{HS}}(8)+4M+4N)\;\; \end{array} \end{array}\right.$$

where $CM_{D1}$, $CM_{D2}$, $CM_{D3}$, and $CM_{D4}$ denote the sub-sequences used in the diffusion operations. $Y_{\mathrm{INT}}$ represents the integer chaotic sequences derived from the original chaotic sequences. $h_{\mathrm{HS}}(k)$ denotes its k-th element and is used to determine the starting positions of the extracted sub-sequences.

Step 2: Row diffusion is then carried out. For each component, based on the index matrix $CM_{D3_{idx}}$ obtained by sorting $CM_{D3}$ in the column direction with a length of 4M, each row of the component is processed. The values of each row are extracted from $CM_{D3_{idx}}$ and denoted as $r_{1}$, $r_{2}$, $r_{3}$, and $r_{4}$. An exclusive-XOR operation is executed among the $r_{1}$-th row of the component, the $r_{2}$-th row of the $CM_{D1}$, and the $r_{3}$-th row of the $CM_{D2}$, with the result stored in the $r_{4}$-th row of the intermediate component. After processing all M rows of $CM_{D3_{idx}}$, the intermediate components are obtained.

Step 3: Column diffusion is subsequently carried out. Based on the index matrix $CM_{D4_{idx}}$ obtained by sorting the $CM_{D4}$ in the row direction with a length of 4N, each column of the components is processed. The values of each column are extracted from $CM_{D4_{idx}}$ and denoted as $c_{1}$, $c_{2}$, $c_{3}$, and $c_{4}$. An exclusive-XOR operation is executed among the $c_{1}$-th column of the components, the $c_{2}$-th column of the $CM_{D1}$, and the $c_{3}$-th column of the $CM_{D2}$, with the result stored in the $c_{4}$-th column of the matrix $CM_{D3}$. After processing all N columns, the diffused component matrices $PI_{R}^{\prime \prime }$, $PI_{G}^{\prime \prime }$, and $PI_{B}^{\prime \prime }$ are obtained.

Step 4: The final encrypted image is constructed by combining the three diffused components as $PI^{\prime \prime }=\{PI_{R}^{\prime \prime },PI_{G}^{\prime \prime },PI_{B}^{\prime \prime })$.

The NECS-IES is completed through the integration of Queen-based permutation and indexed row–column diffusion. The decryption phase follows the inverse operations of the NECS-IES.

## 4. Performance Analysis

**Figure 13:**
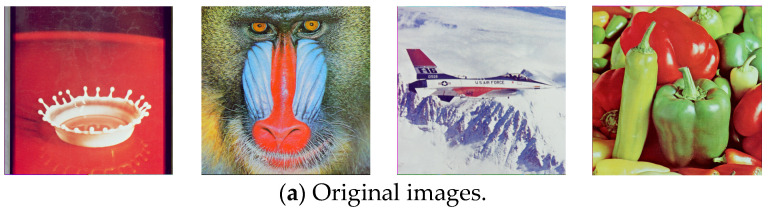

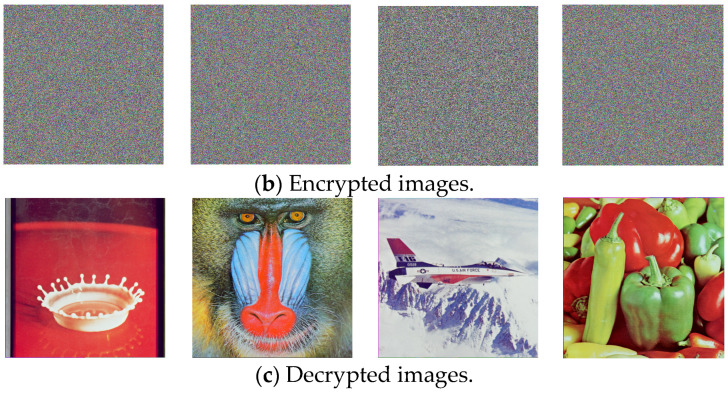
Experimental visualization results.

In this section, a collection of widely used color images is utilized to assess the effectiveness of the NECS-IES as shown in Figure 13. A series of experimental investigations are conducted. Furthermore, multiple representative attack scenarios are applied to the encrypted images to examine the robustness of the NECS-IES.

### 4.1. Key Space

The key is composed of three components: (1) the initial states and control parameters of the 2D–NECS, (2) the hash key $h_{\mathrm{HS}}$ generated from the plaintext image using SHA-512, and (3) a 156-bit binary sequence $k_{R}$. For the 2D–NECS, three parameters (μ, $x_{0}$, $y_{0}$) are employed, and each parameter has a precision of $10^{15}$, resulting in a key space of ${\left(10^{15}\right)}^{3}\approx 2^{150}$. The SHA-512 operation produces a 512-bit output, contributing $2^{512}$ possibilities. In addition, the binary sequence $k_{R}$ introduces $2^{156}$ combinations. Therefore, the key space can be estimated as $2^{150}\times 2^{512}\times 2^{156}=2^{818}$, which is larger than the commonly accepted security threshold of $2^{128}$. It is sufficiently large to effectively resist brute-force attacks [36].

However, it should be noted that the above analysis represents the theoretical key space of the 2D–NECS. In practical implementations, the 2D-NECS is executed using IEEE-754 double-precision arithmetic, where the effective precision of each state variable is constrained by finite floating-point representation. Consequently, the practical cryptographic strength is influenced not only by the theoretical key space but also by finite-precision effects and the dynamical behavior of the CS. To alleviate such degradation, the 2D-NECS incorporates exponential nonlinear terms and a bidirectional coupling structure, which significantly enhances the complexity of the state evolution and reduces the likelihood of short periodic cycles under finite precision. In addition, the plaintext-dependent initialization mechanism based on SHA-512 continuously perturbs the chaotic trajectories for different plaintext images, further mitigating degradation effects. Therefore, although the effective key space is inevitably influenced by digital implementation, it remains substantially larger than the commonly accepted security threshold of $2^{128}$.

### 4.2. Key Sensitivity

Beyond the intrinsic sensitivity of CSs to initial conditions, the robustness of the NECS-IES with respect to key variations is further examined by introducing slight perturbations into the secret key [37]. Specifically, a minimal modification is applied to either the control parameter μ or the hash-derived key component $h_{\mathrm{HS}}$, followed by the encryption and decryption processes.

**Figure 14:**
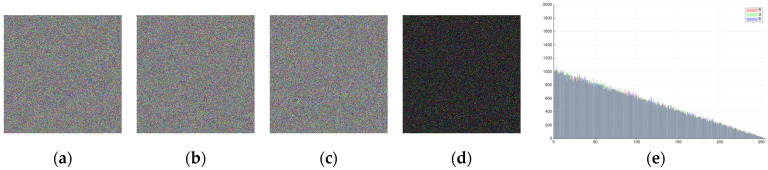
Key sensitivity: the encrypted image using (**a**) the original keys, (**b**) $\mu +10^{-15}$, and (**c**) $h_{\mathrm{HS}}(4)+1$, (**b**–**e**) the histogram of (**d**).

**Figure 15:**
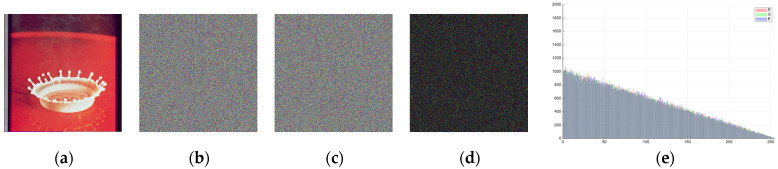
Key sensitivity: the encrypted image using (**a**) the original keys, (**b**) $\mu +10^{-15}$, and (**c**) $h_{\mathrm{HS}}(4)+1$, (**b**–**e**) the histogram of (**d**).

The experimental results presented in Figure 14 reveal that a minute modification of the encryption key produces a dramatically different ciphertext, demonstrating the strong dependence of the NECS-IES on the key parameters. To further evaluate key sensitivity, decryption experiments are performed using keys with slight deviations from the correct one. The results shown in Figure 15 indicate that successful recovery of the plaintext can only be achieved when the exact encryption key is employed, whereas even negligible key mismatches generate completely unintelligible outputs. These observations verify both the reversibility of the decryption and its capability to provide a high level of key sensitivity, which is essential for resisting key-related attacks.

### 4.3. Statical Analyses

#### 4.3.1. Histogram

**Figure 16:**
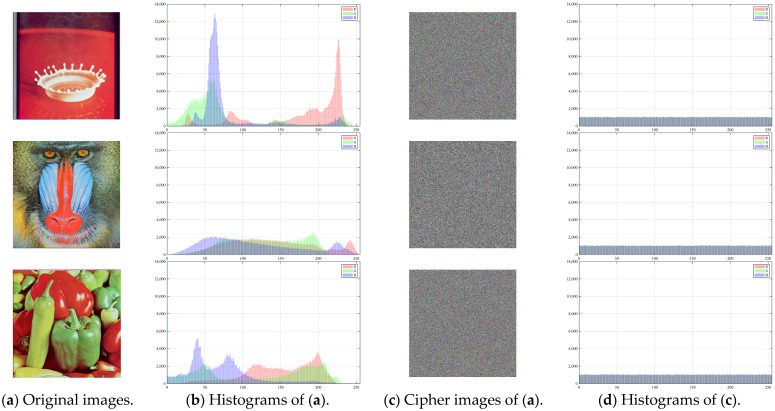
Histograms of the original images and the corresponding encrypted images.

The histogram represents the frequency distribution of pixel intensity values in an image and is widely adopted to evaluate statistical characteristics and resistance to analysis attacks [38]. As tabulated in Figure 16, the histograms of the original images are uneven with noticeable fluctuations and peaks, reflecting inherent redundancies and structural information. In contrast, the histograms of the cipher images become nearly flat and uniformly distributed across all intensity levels. This indicates that the NECS-IES effectively disperses the statistical features of the plaintext, thereby reducing the risk of information leakage and enhancing robustness against histogram-based attacks.

#### 4.3.2. Correlation

This subsection investigates the effectiveness of the NECS-IES by examining the relationship between adjacent pixels in three orientations: horizontal (H), vertical (V), and diagonal (D). The correlation coefficient between neighboring pixel pairs is computed as follows [39]:

(20) $$r_{x,y}=\frac{\sum_{i=1}^{t}\left(x_{i}-E(x))(y_{i}-E(y)\right)}{\sqrt{\sum_{i=1}^{t}{\left(x_{i}-E(x)\right)}^{2}}\sqrt{\sum_{i=1}^{t}{\left(y_{i}-E(y)\right)}^{2}}}$$

where t represents the total number of selected pixel pairs. E(x) and E(y) denote the mean values of the corresponding pixel sequences.

**Figure 17:**
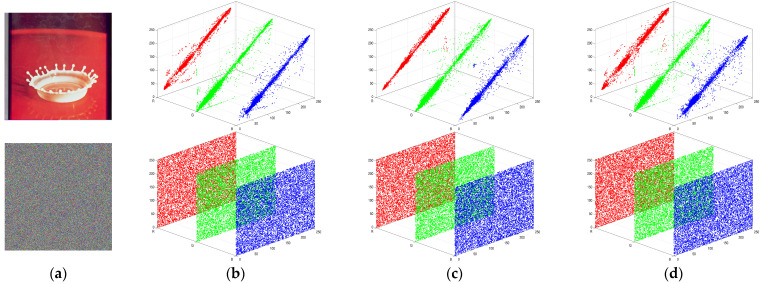
Correlation distributions for plaintext and the corresponding cipher image. (**a**) Original and the corresponding cipher image. (**b**) Horizontal correlation of three channels. (**c**) Vertical correlation of three channels. (**d**) Diagonal correlation of three channels.

According to the results tabulated in Table 2 and Figure 17, the original image shows a high level of correlation among neighboring pixels, whereas the encrypted image exhibits correlation coefficients that are approximately zero in all directions. This indicates that the NECS-IES successfully disrupts the inherent spatial dependency of image data, thereby enhancing resistance to statistical analysis. Furthermore, the correlation coefficients of the encrypted images remain close to zero across all RGB channels, indicating that the proposed scheme effectively removes statistical dependencies among adjacent pixels. The negligible residual correlations further suggest that the ciphertext images reveal little useful information about the underlying plaintext structures.

#### 4.3.3. Information Entropy

Information entropy is a metric used to quantify the uncertainty of a data source [40]. In IESs, it reflects the randomness of the ciphertext and serves as an important indicator of resistance to statistical attacks. For an 8-bit grayscale image with 256 possible intensity levels, the maximum entropy value is 8, which corresponds to a perfectly uniform distribution of pixel values. The entropy is defined as [41]:

(21) $$H(x)=-\sum_{i=1}^{n}P(x_{i})\log_{2}(P(x_{i}))$$

where n denotes the total number of pixels and $P(x_{i})$ represents the probability of occurrence of gray level $x_{i}$. The experimental results tabulated in Table 3 show that the entropy values of the ciphertext are consistently very close to the theoretical maximum score, indicating a high degree of randomness. Furthermore, all test images yield entropy values greater than 7.9992, confirming the robustness of the NECS-IES across different image contents. The similar entropy values observed in the R, G, and B channels indicate a highly uniform ciphertext distribution with minimal statistical redundancy.

### 4.4. Differential Attack

Differential attacks analysis is employed to evaluate how sensitively an IES responds to slight modifications in the input image or key, and to verify whether it exhibits a desirable avalanche property. An effective IES should generate significantly different ciphertexts even when only a single pixel in the plaintext is altered. In this context, two widely used criteria, namely the Number of Pixel Change Rate (NPCR) and the Unified Average Changing Intensity (UACI) [42], are adopted and computed as follows:

(22) $$NPCR=\frac{\sum_{i=1}^{M}\sum_{j=1}^{N}D(i,j)=\left\{\begin{array}{cc} 1 & \mathrm{if}\;c_{1}(i,j)\neq c_{2}(i,j)\\ 0 & \mathrm{if}\;c_{1}(i,j)=c_{2}(i,j) \end{array}\right.}{M\times N}\times 100\%$$

(23) $$UACI=\frac{1}{M\times N}\left(\sum_{i=1}^{M}\sum_{j=1}^{N}\frac{\left|c_{1}(i,j)-c_{2}(i,j)\right|}{255}\right)\times 100\%$$

where $c_{1}(i,j)$ and $c_{2}(i,j)$ denote the pixel values at position (i,j) in two ciphertext images obtained from slightly different plaintexts. NPCR reflects the ratio of pixels that differ between the two encrypted images, while UACI measures the average intensity variation among those pixels. For ideally random images, the expected NPCR and UACI values are approximately 99.6094% and 33.4635%, respectively. Table 4 tabulates the obtained entropy values.

As reported in Table 4, the calculated NPCR and UACI scores are very close to these theoretical benchmarks. This indicates that the NECS-IES possesses a strong encryption mechanism, allowing minor perturbations in the plaintext to spread rapidly throughout the entire ciphertext. Furthermore, the NPCR values consistently exceed 99.60% and the UACI values remain close to the ideal value of 33.46% for all test images and color channels, demonstrating the stability of the NECS-IES under different image contents. The highly consistent results across the R, G, and B channels further verify the effectiveness of the diffusion process in amplifying plaintext variations. Consequently, the NECS-IES exhibits excellent resistance to differential attacks.

### 4.5. NIST Test for Encrypted Images

To further evaluate the randomness of the ciphertext generated by the NECS–IES, the NIST SP800-22 test suite is applied to the encrypted images. The ciphertexts are converted into binary sequences and subjected to the same fifteen tests described in Section 2.1.8. As reported in Table 5, all *p*-values exceed the significance threshold of 0.01, and the pass rates of the non-overlapping template matching, random excursions, and random excursions variant tests satisfy the required criteria. Moreover, the ciphertext sequences exhibit randomness characteristics comparable to those of the original chaotic sequences, indicating that the permutation–diffusion process effectively preserves the randomness of the 2D-NECS. Therefore, the NIST results further confirm that the proposed NECS-IES can generate statistically random ciphertexts with strong security properties.

### 4.6. Chosen-Plaintext and Known-Plaintext Attacks

**Figure 18:**
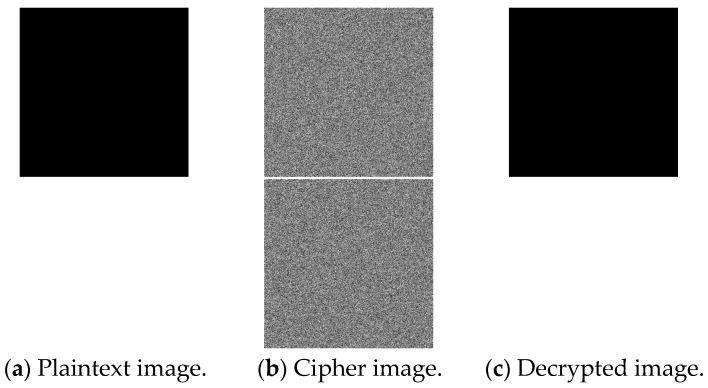
Encryption and decryption results of all-black and all-white images.

To assess the robustness of the NECS-IES against known-plaintext and chosen-plaintext attacks, special plaintext images, including all-black, all-white, and single-pixel-modified images, were employed as shown in Figure 18. Due to the SHA-256-based plaintext-dependent key generation mechanism, different plaintexts produce distinct chaotic sequences and diffusion keys. Moreover, the ciphertexts generated from these special plaintexts exhibit random noise-like appearances and achieve NPCR and UACI values close to the theoretical benchmarks, confirming the strong resistance of the proposed scheme to both attack models as shown in Table 6.

### 4.7. Noise and Data Crop Attacks

**Figure 19:**
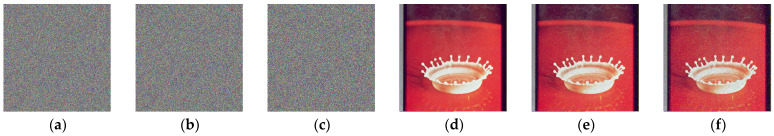
Noise-attack results: (**a**) 0.01, (**b**) 0.05, and (**c**) 0.1 salt and pepper noise intensities of the encrypted image; (**d**–**f**) are the decrypted images of (**a**–**c**).

**Figure 20:**
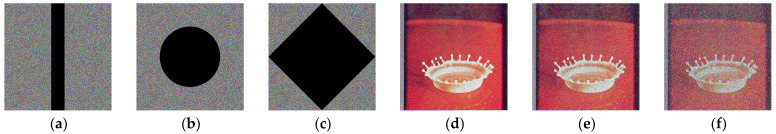
Crop-attack results: (**a**) 1/16, (**b**) 1/4, and (**c**) 1/2 of the cipher image, and (**d**–**f**) are the reconstructed images of (**a**–**c**).

For an IES, the encrypted images are expected to maintain stability when subjected to distortions such as noise interference and partial data removal during transmission or storage. In this work, the robustness of the NECS–IES is evaluated under two typical conditions: salt and pepper noise with varying intensities and cropping attacks with different ratios. The experimental results shown in Figure 19 and Figure 20 indicate that, despite the presence of noticeable degradation caused by noise contamination and missing image regions, the decoded images are still able to retain the essential structural characteristics and recognizable visual content, demonstrating the strong resilience of the NECS-IES.

### 4.8. Computational Complexity

The overall computational complexity of the NECS-IES mainly arises from three parts: key generation, Queen-based permutation, and indexed row–column diffusion. During the key generation stage, the 2D-NECS is iterated to produce sequences of length proportional to 3×M×N, and the hash computation also scales linearly with the image size, leading to a complexity of O(MN). In the permutation stage, three label matrices are constructed and used to reorganize pixel positions across RGB channels. Although nested loops are involved in label generation, each pixel is visited a constant number of times, and the subsequent sorting and reassignment operations are also proportional to the total number of pixels. Therefore, this stage requires O(MN) operations. For the diffusion stage, both row-wise and column-wise processes consist of XOR operations and index-based rearrangements. Since each pixel participates in a fixed number of arithmetic operations, the computational cost remains linear with respect to the image size, i.e., O(MN). Consequently, the total complexity of the NECS-IES is O(MN), which is suitable for IESs.

### 4.9. Discussion

To evaluate the overall performance of the NECS-IES, a comprehensive comparison with several existing IESs is tabulated in Table 7. It can be observed that the NECS–IES achieves the lowest correlation value, which indicates a more effective elimination of statistical dependencies among adjacent pixels. In terms of information entropy, the NECS–IES reaches 7.9995, which is closer to the ideal value of 8 compared with the other IESs, demonstrating superior randomness of the encrypted image. For differential attack metrics, the NPCR value is very close to theoretical expectation and comparable to the best-performing references, while the UACI value is also highly consistent with the ideal value, indicating strong sensitivity to plaintext changes. Overall, the NECS–IES exhibits a balanced and competitive performance across all evaluation metrics, achieving lower correlation, higher entropy, and robust resistance against differential attacks, thereby demonstrating its effectiveness and superiority over the compared IESs.

## 5. Conclusions

The NECS-IES integrates a two-dimensional nonlinear exponential CS with a Queen-driven permutation and index row–column diffusion operations, achieving both strong dynamical behavior and reliable encryption performance. The generated chaotic sequences exhibit high sensitivity and complexity, which are further exploited to construct adaptive permutation indices and diffusion parameters. Through the introduction of a Queen-based permutation mechanism guided by dynamically generated label matrices, the spatial correlation among pixels is effectively disrupted while enabling flexible cross-channel scrambling. In combination with the indexed row–column diffusion process, local pixel variations can rapidly propagate across the entire image, significantly enhancing the overall diffusion capability. Extensive experimental evaluations demonstrate that the NECS-IES provides strong resistance against a variety of attacks, including statistical and differential analyses. The ciphertext images exhibit high randomness and near-zero correlation, confirming the effectiveness of the NECS-IES. Meanwhile, the NECS-IES maintains a reasonable computational cost, making it suitable for secure image encryption.

## Figures and Tables

**Table 1:** NIST test results for chaotic sequences.

NIST Test Item	$p_{x}$	$p_{y}$
Frequency	0.6921	0.4099
Block Frequency	0.7627	0.6467
Runs	0.2793	0.4961
Longest Run	0.0133	0.2700
Rank	0.6414	0.4652
FFT	0.5694	0.6073
Non-overlapping Template	0.8863	0.7334
Overlapping Template	0.6639	0.4448
Universal	0.0988	0.4868
Linear Complexity	0.5353	0.1189
Serial Test *p*-value 1	0.1497	0.4894
Serial Test *p*-value 2	0.5799	0.2851
Approximate Entropy	0.0929	0.7075
Random Excursions	0.3320	0.0917
Random Excursions Variant	0.5446	0.1085
Cumulative Sums-forward	0.9650	0.4377

**Table 2:** Correlation coefficient results.

Test Image	Horizontal			Vertical			Diagonal		
	R	G	B	R	G	B	R	G	B
4.2.01	0.0002	−0.0002	0.0017	0.0004	0.0038	−0.0006	0.0002	−0.0018	0.0016
4.2.03	−0.0024	−0.0017	0.0008	0.0016	0.0017	0.0002	−0.0019	−0.0025	−0.00001
4.2.05	−0.0033	−0.0012	0.0011	−0.0016	−0.0003	−0.0014	−0.0019	0.0013	−0.0009
4.2.06	0.0021	0.0002	0.0014	−0.0005	−0.0004	0.0015	−0.0010	−0.0001	0.0034
4.2.07	0.0020	−0.0002	−0.0004	0.0001	−0.0020	−0.0013	−0.0038	−0.0011	−0.00001
2.2.01	0.0004	0.0008	−0.0009	−0.00003	−0.0008	0.0006	0.0017	0.0006	0.0010
2.2.02	0.0011	0.0004	−0.00007	−0.0011	−0.0003	−0.0014	−0.0005	−0.0008	−0.0013
2.2.03	0.0020	−0.00001	0.0005	0.0001	−0.0001	−0.0009	0.0007	−0.0004	0.0006

**Table 3:** Entropy values.

Test Image	R	G	B	Test Image	R	G	B
4.2.01	7.9993	7.9993	7.9994	4.2.07	7.9992	7.9995	7.9993
4.2.03	7.9992	7.9993	7.9993	2.2.01	7.9998	7.9998	7.9998
4.2.05	7.9993	7.9994	7.9992	2.2.02	7.9998	7.9998	7.9998
4.2.06	7.9992	7.9993	7.9993	2.2.03	7.9998	7.9998	7.9998

**Table 4:** NPCR and UACI values.

Test Image	NPCR (%)			Test Image	UACI (%)		
	R	G	B		R	G	B
4.2.01	99.6037	99.6105	99.6075	4.2.01	33.4335	33.5161	33.4815
4.2.03	99.6132	99.6239	99.6258	4.2.03	33.4288	33.4760	33.4870
4.2.05	99.6208	99.6033	99.6201	4.2.05	33.4741	33.4528	33.4552
4.2.06	99.6117	99.6017	99.6170	4.2.06	33.4457	33.4476	33.4575
4.2.07	99.6132	99.6090	99.6059	4.2.07	33.4572	33.4557	33.4918
2.2.01	99.6026	99.6094	99.6003	2.2.01	33.4391	33.4712	33.4371
2.2.02	99.6081	99.5941	99.6074	2.2.02	33.4534	33.4748	33.4872
2.2.03	99.6121	99.6021	99.6024	2.2.03	33.4781	33.4692	33.4566

**Table 5:** NIST test results of the cipher images.

NIST Test Item	Cipher Image								
	4.2.01			4.2.03			4.2.05		
	R	G	B	R	G	B	R	G	B
Approximate Entropy	0.5108	0.6753	0.7748	0.6900	0.4905	0.3778	0.2590	0.9689	0.3370
Block Frequency	0.4503	0.4473	0.4688	0.3789	0.3675	0.3232	0.7347	0.7067	0.3497
Cumulative Sums-forward	0.7620	0.9960	0.2087	0.2111	0.8820	0.3647	0.4521	0.5407	0.8399
Cumulative Sums-reverse	0.9111	0.9672	0.2910	0.1649	0.9888	0.8182	0.4144	0.7945	0.8643
FFT	0.9535	0.9707	0.6675	0.0434	0.7814	0.6675	0.4130	0.4276	0.3112
Frequency	0.8597	0.9044	0.2795	0.2248	0.8337	0.59111	0.3477	0.7813	0.9769
Linear Complexity	0.4940	0.8967	0.1984	0.1940	0.7776	0.2735	0.0907	0.8880	0.9073
Longest Run	0.4298	0.0964	0.1964	0.3131	0.8033	0.8739	0.0835	0.9793	0.8471
Non-overlapping Template	147/148	148/148	147/148	147/148	146/148	148/148	147/148	145/148	148/148
Overlapping Template	0.1985	0.3374	0.7612	0.2082	0.5630	0.2975	0.0632	0.1332	0.8082
Random Excursions	7/8	7/8	8/8	8/8	8/8	-	8/8	8/8	8/8
Random Excursions Variant	18/18	18/18	18/18	18/18	17/18	-	16/18	18/18	18/18
Rank	0.6323	0.7582	0.2138	0.4759	0.8061	0.1340	0.4896	0.0456	0.8677
Runs	0.3477	0.2360	0.2352	0.7605	0.6387	0.7815	0.9103	0.6625	0.8488
Serial Test *p*-value 1	0.7351	0.5189	0.2607	0.0318	0.7673	0.6604	0.2680	0.0772	0.8252
Serial Test *p*-value 2	0.5151	0.8764	0.3771	0.6872	0.5431	0.7362	0.3813	0.0285	0.9794
Universal	0.1086	0.1712	0.8183	0.6666	0.0437	0.0337	0.2993	0.3330	0.1019

**Table 6:** Correlation, information entropy, NPCR, and UACI results for ciphertext images generated from different chosen plaintexts.

Test Image	Correlation			Entropy	NPCR (%)	UACI (%)
	Horizontal	Vertical	Diagonal			
Pure black	−0.0006	−0.0019	0.0007	7.9993	99.6056	33.4651
Pure white	−0.0012	−0.0040	0.0006	7.9993	99.6040	33.4960

**Table 7:** Comparative analysis for different IESs.

Test Image	Correlation	Entropy	NPCR (%)	UACI (%)
Ref. [43]	0.0755	7.9993	99.6014	33.4798
Ref. [44]	0.0318	7.9985	99.6189	33.4658
Ref. [45]	0.0017	7.9993	99.6118	33.4619
NECS–IES	0.0011	7.9995	99.6094	33.4636

## Data Availability

No new data were created or analyzed in this study.
